# N-cadherin Alleviates Apoptosis and Senescence of Nucleus Pulposus Cells via Suppressing ROS-dependent ERS in the Hyper-osmolarity Microenvironment

**DOI:** 10.7150/ijms.90591

**Published:** 2024-01-01

**Authors:** Luetao Zou, Yiyang Wang, Yanzhu Hu, Liehua Liu, Lei Luo, Zhao Chen, Yunyun Zhuo, Pei Li, Qiang Zhou

**Affiliations:** Department of Orthopedics, The Third Affiliated Hospital of Chongqing Medical University, Chongqing 400010, China

**Keywords:** N-cadherin, nucleus pulposus cells, intervertebral disc degeneration, endoplasmic reticulum stress, hyper-osmolarity microenvironment

## Abstract

The in-situ osmolarity is an important physicochemical factor that regulates cell fate of nucleus pulposus cells (NPCs). Our previous studies demonstrated that reduced N-cadherin (NCDH) expression in nucleus pulposus cells is associated with cellular damage under hyper-osmolarity microenvironment. This study was aimed at exploring the impacts of NCDH on senescence and apoptosis of NPCs, as well as the potential molecular mechanism. By comparing NPCs from patients with lumbar fractures and lumbar disc herniation, we identified a correlation between decreased NCDH expression and increased endoplasmic reticulum stress (ERS), resulting in undesirable cell fate (senescence and apoptosis). After blocking Reactive oxygen species (ROS) or ERS, it was indicated that hyper-osmolarity microenvironment induced ERS was ROS-dependent. Further results demonstrated the correlation in rat NPCs. Upregulation of NCDH expression reduced ROS-dependent ERS, thus limiting undesirable cell fates *in vitro*. This was further confirmed through the rat tail acupuncture injection model. NCDH overexpression successfully mitigated ERS, preserved extracellular matrix production and alleviating intervertebral disc degeneration *in vivo*. Together, NCDH can alleviate senescence and apoptosis of NPCs by suppressing ROS-dependent ERS via the ATF4-CHOP signaling axis in the hyper-osmolarity microenvironment, thus highlighting the therapeutic potential of NCDH in combating degenerative disc diseases.

## Introduction

As the population ages, low back pain has been a commonly seen condition that affects the daily activities of middle-aged and senior individuals, resulting in significant economic burdens on society 1. Intervertebral disc degeneration (IDD) is a critical element precipitating the onset of low back pain 2. Up to now, relevant mechanism of IDD is not clear. Researches have shown that this complex degenerative mechanism resulted from microenvironmental changes of intervertebral disc tissue is mediated by various etiologies, such as natural aging, smoking, mechanical stress, spine infection, spine trauma and heredity 3.

Osmotic pressure is one of the main microenvironmental factors during IDD 4. In vivo, various forms of compressive stress can squeeze the liquid out of the nucleus pulposus (NP) tissue and resultantly increase the in-situ osmotic pressure in intervertebral disc 5. Recent studies have shown that the hyper-osmolarity microenvironment significantly activates intracellular reactive oxygen species (ROS) in nucleus pulposus cells (NPCs) 6, 7. According to the free radical theory, ROS closely relates with the attenuation of tissue and organ function, including the occurrence and progression of IDD 8-10. Early studies suggested that ROS are effective proapoptotic factors 10, which promotes apoptosis through the classical mitochondrial apoptosis pathway 11. In addition, ROS are the basic stimulators of senescence and really promote senescence in human NPCs 9, 12.

Recent researches have considered that endoplasmic reticulum stress (ERS) is an important downstream event of ROS and regard it as a symbol of cellular damage in some pathological conditions 13, 14. The endoplasmic reticulum is the largest organelle. Disruption of endoplasmic reticulum homeostasis can induce a high ERS level, which is involved in multiple aging-associated diseases 15. Previous studies have shown that high ERS levels promote apoptosis in chondrocytes and that ROS induce sustained ERS in airway smooth muscle cells 16, 17. In the NPCs, advanced glycation end products (AGEs), the acidic microenvironment and inflammatory factors, can significantly activate ERS and promote senescence and apoptosis of NPCs 18-20. In vivo studies have revealed that ERS is present in mechanical imbalance-induced IDD rats and is related to intervertebral disc cell apoptosis and some other degenerative changes 21. ATF4-CHOP axis is one of important pathway to mediate the unfold protein response and ultimately induces ERS 22. Moreover, several studies have also showed that ATF4-CHOP axis-mediated ERS makes a significant role in regulating apoptosis and senescence in other cell types 23, 24. However, whether ROS-dependent ERS via the ATF4-CHOP axis is related to apoptosis and senescence of NPCs under a hyper-osmolarity microenvironment is unclear at present.

N-cadherin (NCDH), a subtype of the cadherin family, is first identified in synaptic formation in the nervous system. Recently, NCDH has been considered a typical surface marker that maintains the phenotype of NPCs when faced with certain pathological factors, and its expression in NPCs is significantly reduced during IDD 25, 26. The downregulation of NCDH is related to apoptosis and senescence in multiple cell types or tissues. For example, the downregulation of NCDH expression can promote apoptosis in cancer cells (i.e. non-small cell lung cancer, tongue squamous carcinoma cells) and non-tumor cells (i.e. vascular smooth muscle cells in atherosclerotic tissues) 27-29. Furthermore, NCDH is a key protein that reduces senescence and apoptosis in NPCs 25. Finally, N-CDH is able to decrease ROS generation and then alleviate senescence of NPCs under a high glucose condition 30. Based on the above exposition, we deduce that NCDH may be an upstream regulator of ROS-dependent ERS and the secondary pathological events in NPCs.

At present, the direct relationship between NCDH expression and cell fates (apoptosis and senescence) of NPCs under a hyper-osmolarity microenvironment is unclear. Based on previous reports and our own experiments, we hypothesize that NCDH may alleviate apoptosis and senescence of NPCs by inhibiting ROS-dependent ERS in a hyper-osmolarity microenvironment. Thus, we designed this study to reveal whether and how NCDH alleviates hyper-osmolarity microenvironment-mediated apoptosis and senescence by inhibiting ROS-dependent ERS in rat NPCs.

## Materials and Methods

### Human NP tissues collection

We collected NP tissue samples from 29 patients (Table S1), including 4 patients with lumbar vertebral fracture (LVF, 4 males, Pfirrmann I-II) and 25 patients with intervertebral disc degeneration (IDD, 11 males and 14 females, Pfirrmann IV-V), following Pfirrmann classification criteria determined through preoperative MRI (magnetic resonance imaging) scans at in the Department of Spine Surgery of Third Affiliated Hospital of Chongqing Medical University.

Inclusion criteria of LVF group: (1) No history of lower back pain prior to admission; (2) Diagnosed with lumbar vertebral fracture based on admission examination; (3) Ruled out other lumbar spine disorders; (4) Strong willingness for surgical treatment and consent to participate in this study. Inclusion criteria of IDD group: (1) The medical history and imaging examinations meet the diagnostic criteria for lumbar disc herniation; (2) Symptoms have not improved after at least 3 months of conservative treatment; (3) Strong willingness for surgical treatment and consent to participate in this study. Exclusion criteria of LVF group and IDD group: (1) Infection in the vertebral body or adjacent area; (2) Confirmed diagnosis of any tumor; (3) Coexisting with other underlying diseases that make the patient not suitable for surgery; (4) Refusal to participate in this study.

### NPCs isolation

Sprague-Dawley (SD) rats (female, 250±5 g, 5-8 weeks old) were received from the Experimental Animal Center of Chongqing Medical University. Rats were humanely sacrificed with excess CO2, and lumbar discs were extracted. The human or rat NP tissues were obtained by corneal scissors and treated with 0.2% type II collagenase (Sigma, USA) for 1-2 h after a 10-minute digestion with 0.25% trypsin (Beyotime, Wuhan, China) at 37 °C. NPCs were subsequently isolated via centrifugation and cultured under standard conditions (37 °C, 5% CO2).

### Rat NPCs culture and treatment

The NPCs were cultured in conventional medium (control group, 330 mOsm/kg) or hyperosmotic medium (experimental group, 550 mOsm/kg). The conventional medium is prepared by adding 10% fetal bovine serum (Gibco, Grand Island, New York, USA) and 1% penicillin/streptomycin (Beyotime, Wuhan, China) to DMEM/F-12 medium (Hyclone, Logan, Utah, USA). The hyperosmotic medium was obtained by addition of NaCl based on the conventional medium. Endoplasmic reticulum stress inhibition: rat NPCs were treated with sodium phenylbutyrate (4-PBA, 2.5 nmol/L, MedChemExpress, Monmouth Junction, New Jersey, USA) in DMEM/F-12 medium for 30 min, then change to hyperosmotic medium for 24 h. ROS inhibition: rat NPCs were treated with N-acetylcysteine (NAC, 1 μmol/L, MedChemExpress, Monmouth Junction, New Jersey, USA) in hyperosmotic medium for 24 h.

### Lentivirus vector transfections

The lentivirus vector regulating NCDH (LV-NCDH and RNAi-NCDH) were purchased from GeneChem (Guangzhou, China). NPCs were seeded in 24-well plates (2×10^4^ cells per well) and transfected with LV-NCDH for 24-36 h, followed by puromycin selection to establish stable cell lines. The transfection efficacy was verified by western blot in this study.

### SiRNA transfections

SiRNA (GeneBiogist, Guangzhou, China, geneID 24525) was dissolved in RNase-free water (40 nM) and stored at 4°C. Lipofectamine 2000 (Beyotime, Wuhan, China) was diluted in F12 medium (diluted at 1:50) and stored at room temperature. The siRNA was mixted with the transfection reagent in the medium. This mixture was added to NPCs. 24 h later, the successfully transfected cells were identified. The siRNA sequences are as follows to target KRAS (P21): KRAS(r)-si-1: GAAUCACUUUGUGGAUGAA tt, KRAS(r)-si-2: CCAUUAUAGAGAACAAAUU tt, KRAS(r)-si-3: GAGAAAUUCGAAAACAUAA tt.

### Senescence-Associated β-Galactosidase (SA-β-Gal) Staining

SA-β-Gal staining is a commonly applied method to detect the cellular senescence. After treatment, NPCs were fixed by 0.2% paraformaldehyde for 15 min at ambient temperature. After being rinsed with phosphate buffer saline (PBS), NPCs were stained with X-gal solution (Beyotime, Wuhan, China) for 24 h at 37 °C. Senescent NPCs were observed with the use of an optical microscope (Olympus, Japan).

### Measurement of intracellular ROS content

The intracellular ROS level was measured by dihydroethidium (DHE) probe staining (X-Y Biotechnology, Shanghai, China). After treatment, NPCs were seeded in 24-well plates and incubated with culture medium supplemented with 10 mg/ml DHE for 30 min after treatment. Then, observation of NPCs was conducted under a fluorescence microscopy (Olympus, Japan). ROS content was indirectly reflected by the intensity of red fluorescence.

### Flow cytometry

To assess apoptosis level, NPCs was stained by the Annexin V-APC/DAPI apoptosis detection kit (Procell, Wuhan, China) after washing NPCs twice with PBS. To assess cell cycle, NPCs were fixed by ice-cold 70% ethanol at 4 °C for 24 h and then stained by NUCLEAR-ID Cell Cycle Kit (Enzo Life Sciences, Farmingdale, New York, USA). Subsequent testing and analysis of apoptosis and cell cycle samples were conducted according to the instructions.

### Western blot

Protein extraction from NP tissue or NPCs (human and rat) was performed using the MinuteTM total protein extraction kit (Invent Biotech, Minneapolis, Minnesota, USA). Equivalent-mass samples were separated on 10% or 12% SDS-PAGE and transferred to PVDF. PVDF membranes were blocked, then incubated with primary antibodies overnight at 4 °C followed by secondary antibody (Beyotime, Wuhan, China) incubation at room temperature for 90 min. Finally, chemiluminescence Kit (Bio-Rad, Hercules, California, USA) was used to visualize protein bands. Antibodies information: Cleaved-Caspase-3 (1:1000, #9661), CHOP (1:1000, #2895), ATF4 (1:1000, #11815), XBP1s (1:1000, #40435) and P-CDK1 (Thr161, 1:1000, #9114) were purchased from CST (Danvers, Massachusetts, USA). NCDH (1:5000, ab76011), P21 (1:1000, ab109199), P53 (1:1000, ab26), Cleaved-PARP1(1:5000, ab32064), BIP (1:1000, ab21685), ATF6 (1:1000, ab37149), P16 (1:10000, ab51243) and CDK1 (1:10000, ab32094) were purchased from Abcam (Cambridge, United Kingdom). β-actin (1:5000, 51067-2-AP), HRP-Goat Anti-Rabbit IgG (1:10000, SA00001-2) and HRP-Goat Anti-Mouse IgG (1:10000, PR30012) were purchased from Proteintech (Wuhan, China).

### Cell Counting Kit-8 (CCK-8) analysis

The Enhanced Cell Counting Kit-8 was purchased from Beyotime (Wuhan, China). The rat NPCs were cultured in the 96-well plate under standard culture conditions (37°C, 5% CO2) for 24 h. Subsequently, the NPCs were treated according to the research design. Upon reaching the intervention time point, the culture medium was removed, and CCK-8 working solution (CCK-8 diluted 1:10 with serum-free culture medium) was directly added to the 96-well plate. The plate was then incubated in the dark under standard conditions for 1 h. Absorbance was measured at a wavelength of 450 nm using a microplate reader (Tecan, Manedorf, Switzerland), and cell viability was assessed based on the experimental objectives.

### Transmission electron microscopy (TEM)

NPC pellets, harvested by centrifugation, were fixed with 2.5% glutaraldehyde for 24 h at 4 °C. Subsequently, they were dehydrated in ethanol, embedded in Epon 812, and sectioned into ultrathin slices. These slices were stained with lead citrate and observed using an electron microscope (Hitachi, Japan).

### Rat tail puncture injection model

The SD rats (female, 250±5 g, 8 weeks old) were purchased from the Experimental Animal Center of Chongqing Medical University and they were randomly assigned to three distinct groups (n=5/group): sham group (Control group), LV-NC group (puncture with LV-NC group) and LV-NCDH group (puncture with LV-NCDH group). The Sham group underwent a sham operation, as controls. The LV-NC group and LV-NCDH group underwent puncture modeling 31 and received an injection of PBS suspension containing LV-NC or LV-NCDH during the puncture. The lentivirus were administered into the rat tail NP tissue according to the procedure employed in a prior investigation 32, 33. No postoperative complications were observed. Four weeks after surgery, MRI examination was performed for radiological evaluation of the intervertebral disc and the target intervertebral disc was taken out for further study. The animal results were reported following the ARRIVE guidelines.

### TUNEL staining

Tissue samples were paraformaldehyde-fixed and parrffin-embedded, and then tissue slices (5 μm thick) were prepared. To perform TUNEL staining assay, tissue slices were sequentially dewaxed and dehydrated by xylene and gradient ethanol. After treating tissue slices with protein K working solution (20 μg/ml) for 30 min at 37 °C and 2% hydrogen peroxide for 5 min at room temperature, TUNEL staining assay was performed by following the manufacture's instruction. DAPI solution was adopted to stain cellular nuclei. Finally, NP tissue observation was conducted under a fluorescence microscope (Olympus, Japan).

### HE staining and immunofluorescence staining for tissue sample

Disc tissues were fixed in 4% paraformaldehyde paraffin-embedded, and sliced into 5 μm-thick sections. After xylene dewaxing, standard HE staining was performed. For immunofluorescence, the samples underwent xylene I and II dewaxing, rehydration in gradient alcohol, and rewatered with gradient alcohol. After antigen repair by citric acid, tissue slices were sequentially blocked with blocking serum (Beijing ZhongShan, China) at 37 °C for 30 min, incubated with the primary antibody (anti-BIP, diluted at 1:100) at 4 °C overnight. Subsequently, the fluorescent secondary antibody was introduced and subjected to incubation within the wet box under ambient conditions for 90 min, and DAPI was supplemented and incubated in the dark for 2 min. Finally, HE staining and immunofluorescence staining were found under a light microscope or a fluorescence microscope.

### Statistical analysis

SPSS software (version 20.0. SPSS Inc, USA) was used for the statistical analyses. The assessment of dissimilarities among groups were executed through one-way analysis of variance (ANOVA). P-value < 0.05 was defined significance. All statistical results were repeated for three times.

## Results

### NP tissue from IDD patients exhibited a lower expression of NCDH and an enhanced occurrence rate of apoptosis and senescence

**Figure 1:**
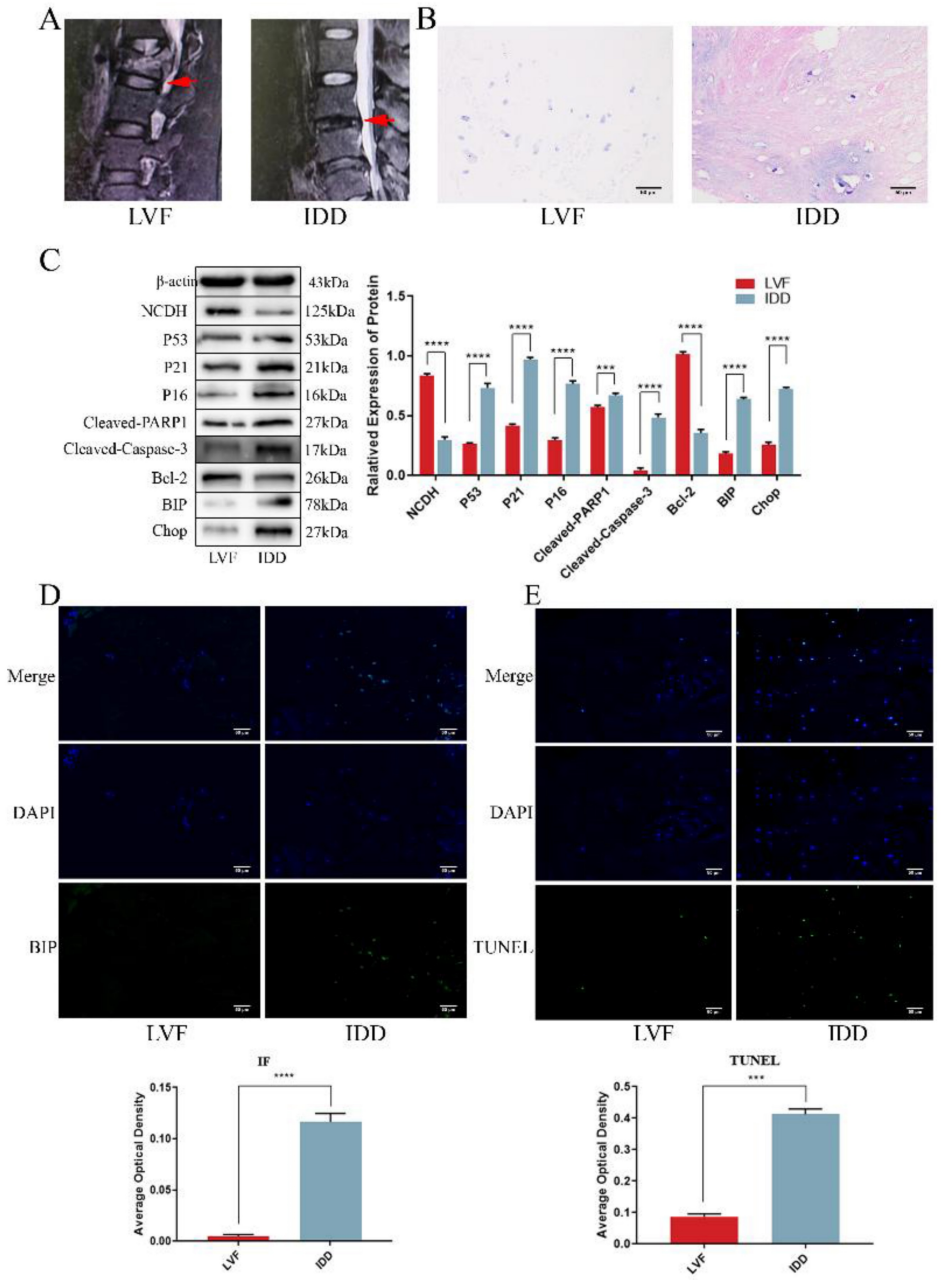
** NP tissue from IDD patients exhibited a lower expression of NCDH and an enhanced occurrence rate of apoptosis and senescence.** (A): Representative lumbar MRI photographs from LVF (Grade I, red arrows) and IDD (Grade IV, red arrows) patients were classified according to the Pfirrmann grading system. (B): HE staining of NP tissue from LVF and IDD patients. Scale bars: 50 μm. (C): Western blot analysis of protein expression of NCDH, P53, P21, P16, Cleaved-PARP1, Cleaved-caspase 3, Bcl-2, BIP and CHOP in NP tissue samples from LVF and IDD patients. (D): Immunofluorescence staining analysis of BIP expression in the NPCs from LVF and IDD patients. Scale bars: 50 μm. (E): TUNEL staining analysis of cell apoptosis of NP tissue from LVF and IDD patients. Scale bars: 50 μm. Note: The data represents mean ± SD (n=3); ***p<0.001, ****p<0.0001 vs. LVF group. LVF: lumbar vertebral fracture, IDD: intervertebral disc degeneration, NP: nucleus pulposus, NPCs: nucleus pulposus cells.

The previous studies highlighted a significant decrease in NCDH expression in degenerative disc samples and its influence on extracellular matrix macromolecules (EMC, such as Aggrecan and Col2a1), yet the underlying mechanism remained unclear 34, 35. Here, we collected clinical disc samples and examined the relationship between the decline in NCDH expression and pathological processes that affect extracellular matrix production, such as apoptosis, senescence and endoplasmic reticulum stress. Structural changes and tissue degeneration were showed in the IDD group using MRI and HE staining (Fig. 1A-B). Western blot, immunofluorescence and TUNEL staining (Fig. 1D-E) showed that the decrease of NCDH expression coincided with the increased ERS level (i.e. increases protein expression of BIP and CHOP, enhanced immunofluorescence staining intensity of BIP), senescence (i.e. elevated protein expression of P53 and P16) and apoptosis (i.e. elevated protein expression of Cleaved-PARP1 and Cleaved-Caspase-3, and enhanced TUNEL-positive staining) in the human NPCs. These results confirmed that senescence and apoptosis occurred simultaneously with the loss of NCDH in degenerated NPCs. Notably, ERS and ROS could activate each other during protein folding imbalance 36, suggesting ROS involvement in multiple cell fates (apoptosis and senescence) during NPCs degeneration.

### Hyper-osmolarity induces undesirable cell fates (senescence and apoptosis) and reduces NCDH expression in rat NPCs

**Figure 2:**
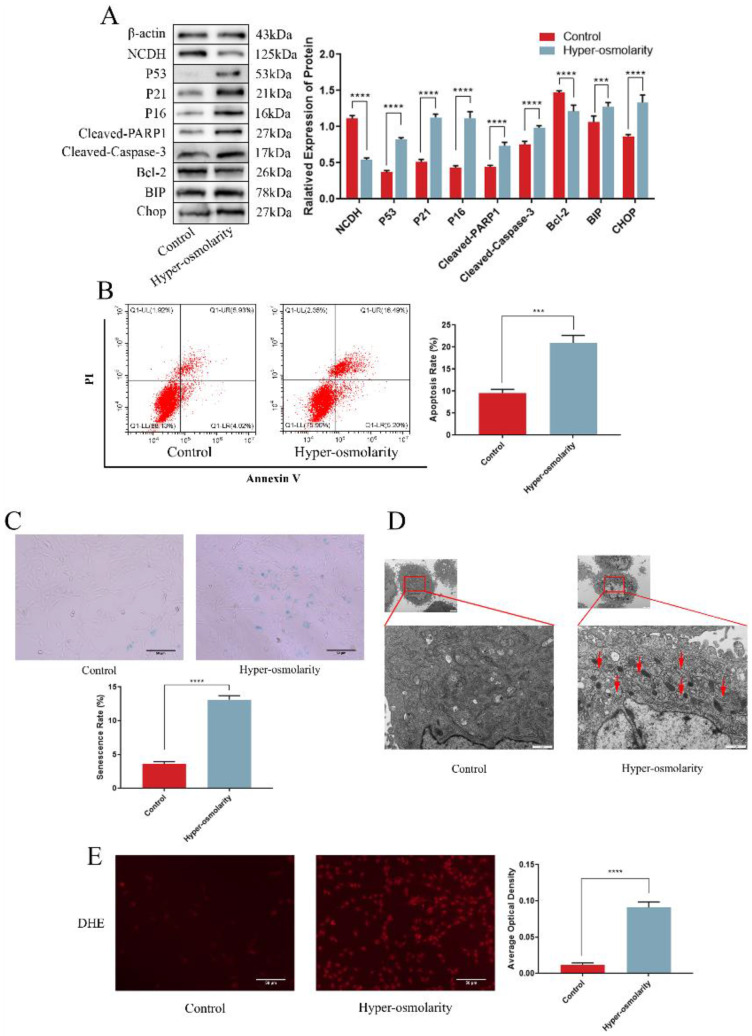
** Hyper-osmolarity induces undesirable cell fates (apoptosis and senescence) and reduces NCDH expression of NPCs.** (A): Western blot analysis of protein expression of NCDH, P53, P21, P16, Cleaved-PARP1, Bcl-2, Cleaved-Caspase-3, BIP and CHOP in NPCs in isotonicity (330 mOsm/kg) or hyper-osmolarity (550 mOsm/kg) culture. (B-C): Flow cytometry and SA-β-Gal staining of NPCs in isotonicity or hyper-osmolarity culture. Scale bars: 50 μm. (D): TEM analysis of ER morphology (red arrow) of NPCs in isotonicity or hyper-osmolarity culture. Scale bars: 1 μm. (E): DHE probe staining analysis of ROS content of NPCs in isotonicity or hyper-osmolarity culture. Scale bars: 50 μm. Note: The data represents mean ± SD (n=3); ***p<0.001, ****p<0.0001 vs. control. NP: nucleus pulposus, NPCs: nucleus pulposus cells, SA-β-Gal: Senescence-Associated β-Galactosidase, TEM: transmission electron microscopy, ROS: reactive oxygen species, DHE: dihydroethidium, ER: endoplasmic reticulum.

According to current reports, we adjusted the osmotic pressure of the medium to 550 mOsm/kg (Fig. S1) with NaCl (analytical purity) to effectively simulate the hyper-osmolarity microenvironment 6. Western blot analysis suggested that the expression of NCDH was notably reduced in the hyper-osmolarity group, whereas the senescence proteins (P53 and P16) and the apoptotic proteins (Cleaved-PARP1 and Cleaved-Caspase-3) were obviously increased, and the anti-apoptotic protein Bcl-2 was decreased (Fig. 2A). In relative to the control group (330 mOsm/kg, the normal osmotic pressure of conventional medium), flow cytometry results showed a significantly higher apoptosis rate in the hyper-osmolarity group, as well as an elevated senescence rate indicated by the SA-β-Gal staining (Fig. 2B-C). These results indicated that the hyper-osmolarity decreased NCDH expression, and promoted senescence and apoptosis of NPCs.

We also explored the profile of ROS generation and ERS in NPCs in the hyper-osmolarity microenvironment due to the increased expression of the ERS marker (BIP) in degenerative disc samples and the potential correlation between ERS and ROS. Through comparison with the control group, western blot analysis demonstrated that the ERS marker (BIP and CHOP) expression was significantly elevated in the hyper-osmolarity group (Fig. 2A). For endoplasmic reticulum morphology, TEM results indicated that the NPCS in the hyper-osmolarity group manifested a distended and inflated endoplasmic reticulum (red arrow) in relative to the control group (Fig. 2D). Additionally, the DHE probe staining assay demonstrated that ROS generation in the hyper-osmolarity group was increased in relative to the control group (Fig. 2E). Similarly, ROS and ERS formed a vicious cycle that activates apoptosis and senescence in other types of cells 13, 37. These findings indicated that ROS and ERS might be involved in senescence and apoptosis of NPCs in the hyper-osmolarity microenvironment.

### Hyper-osmolarity induces cellular undesirable cell fates (senescence and apoptosis) in NPCs through ROS-dependent ERS

**Figure 3:**
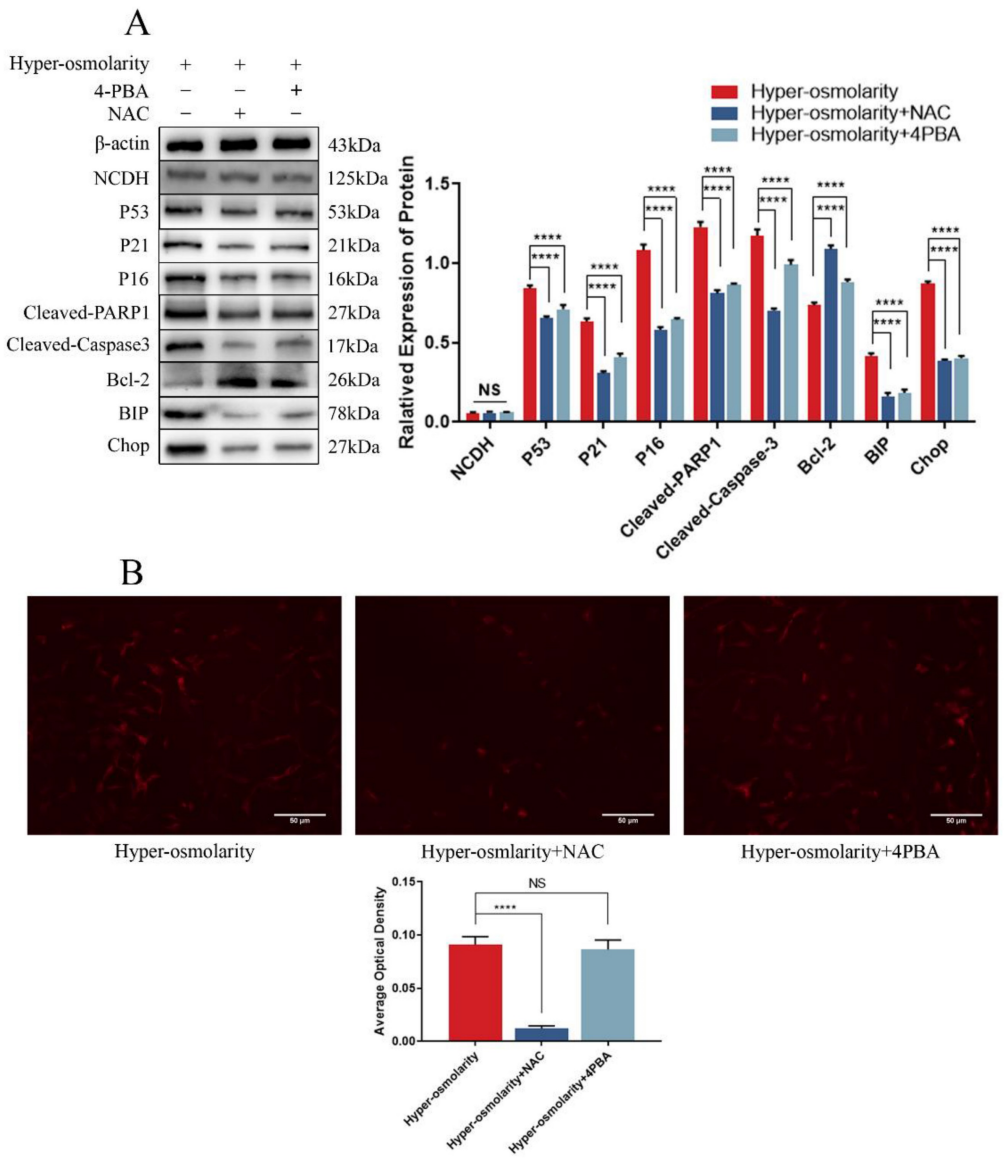
** Hyper-osmolarity induces undesirable cell fates (apoptosis and senescence) of NPCs through ROS-dependent ERS.** (A): Western blot analysis of expressions of NCDH, P53, P21, P16, Cleaved-PARP1, Cleaved-Caspase-3, Bcl-2, BIP and CHOP. (B): DHE staining analysis of ROS content. Scale bars: 50 μm. Note: The data represents mean ± SD (n=3); NS>0.05, ****p<0.0001 vs. hyper-osmolarity group. NPCs: nucleus pulposus cells, ROS: reactive oxygen species, DHE: dihydroethidium.

To understand the relationship between ERS and ROS in hyper-osmolarity microenvironment, we applied NAC to inhibit ROS, 4-PBA to block ERS and observe how other cellular functional changes. Western bolt (Fig. 3A) showed that ERS, apoptosis and senescence of NPCs in the hyper-osmolarity were significantly attenuated after addition of NAC or 4-PBA, which was indicated by the down-regulation of ERS markers (BIP and CHOP), senescence markers (P53 and P16) and pro-apoptotic markers (Cleaved-Caspase-3 and Cleaved-PARP1), and up-regulation of anti-apoptotic marker (Bcl-2). However, DHE probe staining showed that there was no remarkable change in the ROS generation before and after 4-PBA supplementation, whereas ROS generation was significantly decreased after NAC supplementation in the hyper-osmolarity environment (Fig.3B), indicating that ERS-induced NPCs senescence and apoptosis in the hyper-osmolarity environment may be ROS-dependent. Furthermore, the expression of NCDH was not affected by either 4-PBA or NAC (Fig. 3A), suggesting that NCDH may be an upstream regulator of ROS-dependent ERS in NPCs in the hyper-osmolarity environment.

### NCDH overexpression alleviates cellular undesirable cell fates (senescence and apoptosis) in rat NPCs under hyper-osmolarity microenvironment

**Figure 4:**
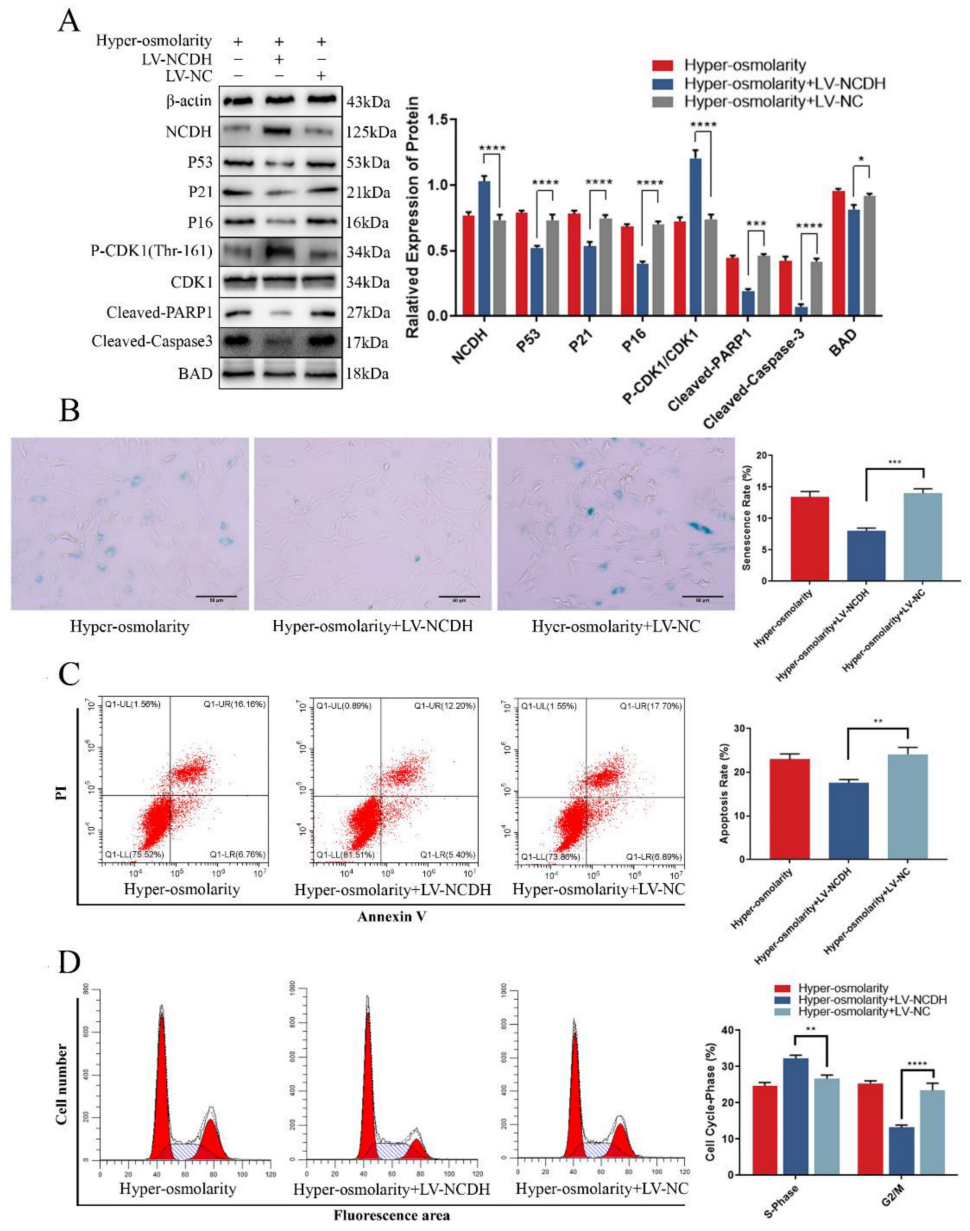
** NCDH overexpression alleviates cellular undesirable cell fates (cell senescence and apoptosis) of NPCs in the hyper-osmolarity microenvironment.** (A): Western bolt analysis of protein expression of NCDH, P53, P21, P16, Cleaved-PARP1, Cleaved-Caspase 3 and p-CDK1 (Thr-161) in NPCs after NCDH overexpression in the hyper-osmolarity culture. (B): SA-β-Gal staining analysis of cell senescence rate in NPCs. Scale bars: 50 μm. (C-D): Flow cytometry analysis of cell apoptosis and cell cycle in NPCs. Note: The data represents mean ± SD (n=3); **p<0.01, ***p<0.001, ****p<0.0001 vs. hyper-osmolarity with LV-NC group. NPCs: nucleus pulposus cells, SA-β-Gal: senescence-Associated β-Galactosidase.

NCDH, which is a phenotypic maintenance marker of NPCs 26, inhibits apoptosis and senescence in a variety of tissues and cells 27-29. To explore the role of NCDH in the hyper-osmolarity environment, we used lentivirus (LV-NCDH) to estimate whether NCDH overexpression (NCDH expression is too low in the hyper-osmolarity microenvironment to further knock down, Fig S2) could protect NPCs against the hyper-osmolarity microenvironment-induced undesirable cell fates (apoptosis and senescence, Figure 4A). In this study, CCK-8 analysis, flow cytometry and SA-β-Gal staining showed that LV-NCDH could significantly enhance the cell viability (Fig. S3B) and reduce apoptosis, G2/M phase arrest and senescence under a hyper-osmolarity condition (Fig. 4B-D). These results revealed that the enhancing NCDH expression reversed apoptosis and senescence of NPCs in a hyper-osmolarity microenvironment.

A novel study reported that P21 was a common target for glioblastoma cells to activate apoptosis and senescence through cell cycle regulation and P21-Bad axis 38. Our western blot results demonstrated that after transfection with LV-NCDH, the high expression of P21 and Cleaved-PARP1 induced by the hyper-osmolarity microenvironment was inhibited, and at the same time, the expression of Bad was decreased (Fig. 4A). Similarly, phosphorylation level of the classical cell cycle regulator CDK1 (Thr-161) increased while P21 expression decreased (Fig. 4A). Thus, we hypothesize that P21 may be the potential target for NCDH to alleviate the fates of undesirable cell (senescence and apoptosis) in NPCs mediated by hyper-osmolarity microenvironment.

### P21 is the critical trigger to regulate apoptosis and senescence in NPCs under hyper-osmolarity microenvironment

**Figure 5:**
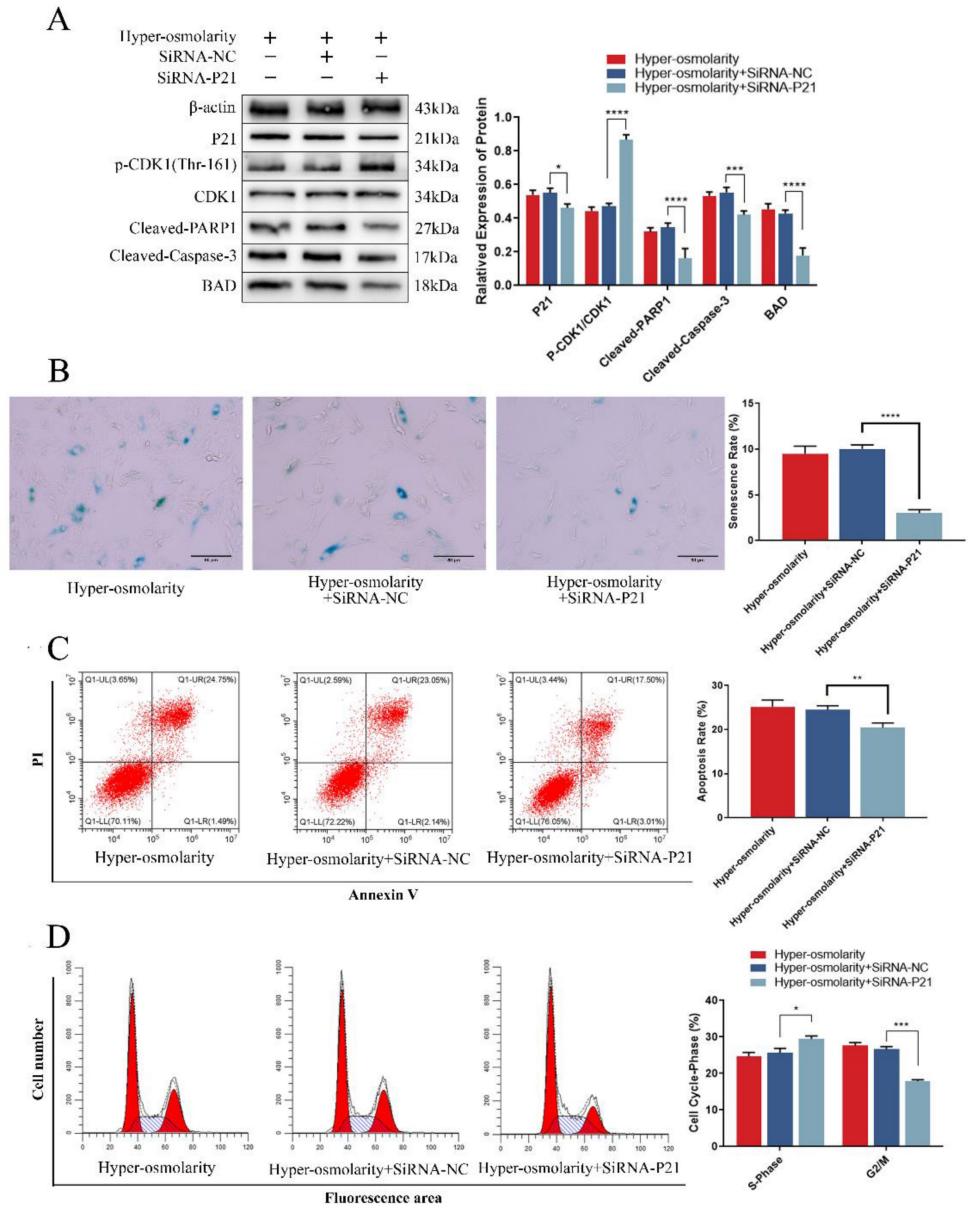
** P21 is the critical trigger to regulate apoptosis and senescence in NPCs under hyper-osmolarity microenvironment.** (A): Western bolt analysis of protein expression of P21, BAD, Cleaved-Caspase-3, Cleaved-PARP1 and p-CDK1 (Thr-161) in NPCs with or without SiRNA treatment. (B): SA-β-Gal staining analysis of cell senescence rate in NPCs. Scale bars: 50 μm. (C-D): Flow cytometry analysis of cell apoptosis and cell cycle in NPCs. Note: The data represents mean ± SD (n=3); *p<0.05, **p<0.01, ***p<0.001, ****p<0.0001 vs. hyper-osmolarity with siRNA-P21 group. NPCs: nucleus pulposus cells, SA-β-Gal: senescence-Associated β-Galactosidase.

To further investigate the relationship between P21 and undesirable cell fate of NPCs, we analyzed the changes of apoptosis and senescence and the expression of relevant markers after transfection of SiRNA-P21 under a hyper-osmolarity microenvironment. The results showed that down-regulating of P21 expression reduced the high expression of Bad and apoptotic protein (Cleaved-Caspase-3, Cleaved-PARP1) mediated under the hyper-osmolarity microenvironment in NPCs (Fig. 5A). Meanwhile, the flow cytometry showed that apoptotic cell rates decreased significantly (Fig. 5C). It has been reported that the P21-Bad axis plays an important role in inducing apoptosis 39, 40. Furthermore, SiRNA-P21 transfection promoted CDK1 phosphorylation (Thr-161) level (Fig. 5A) and cell viability (Fig. S3C) while reducing cell cycle arrest (Fig. 5B) and senescence (Fig. 5D). In eukaryotic cells, inhibition of CDK1 phosphorylation (Thr-161) by P21 is considered to be a key process in inducing G2/M phase arrest 38, 41 and a series of studies have shown that cell cycle arrest induces senescence of NPCs 42, 43, suggesting that P21-regulated cell cycle arrest may participate in NPCs degeneration. These results suggested that NCDH overexpression could attenuate undesirable cell fates (apoptosis and senescence) of NPCs in hyper-osmolarity microenvironment, and P21 may be a key target in this process.

### ATF4-CHOP axis is a key signaling transduction pathway behind the protective effects of NCDH overexpression in rat NPCs under the hyper-osmolarity microenvironment

**Figure 6:**
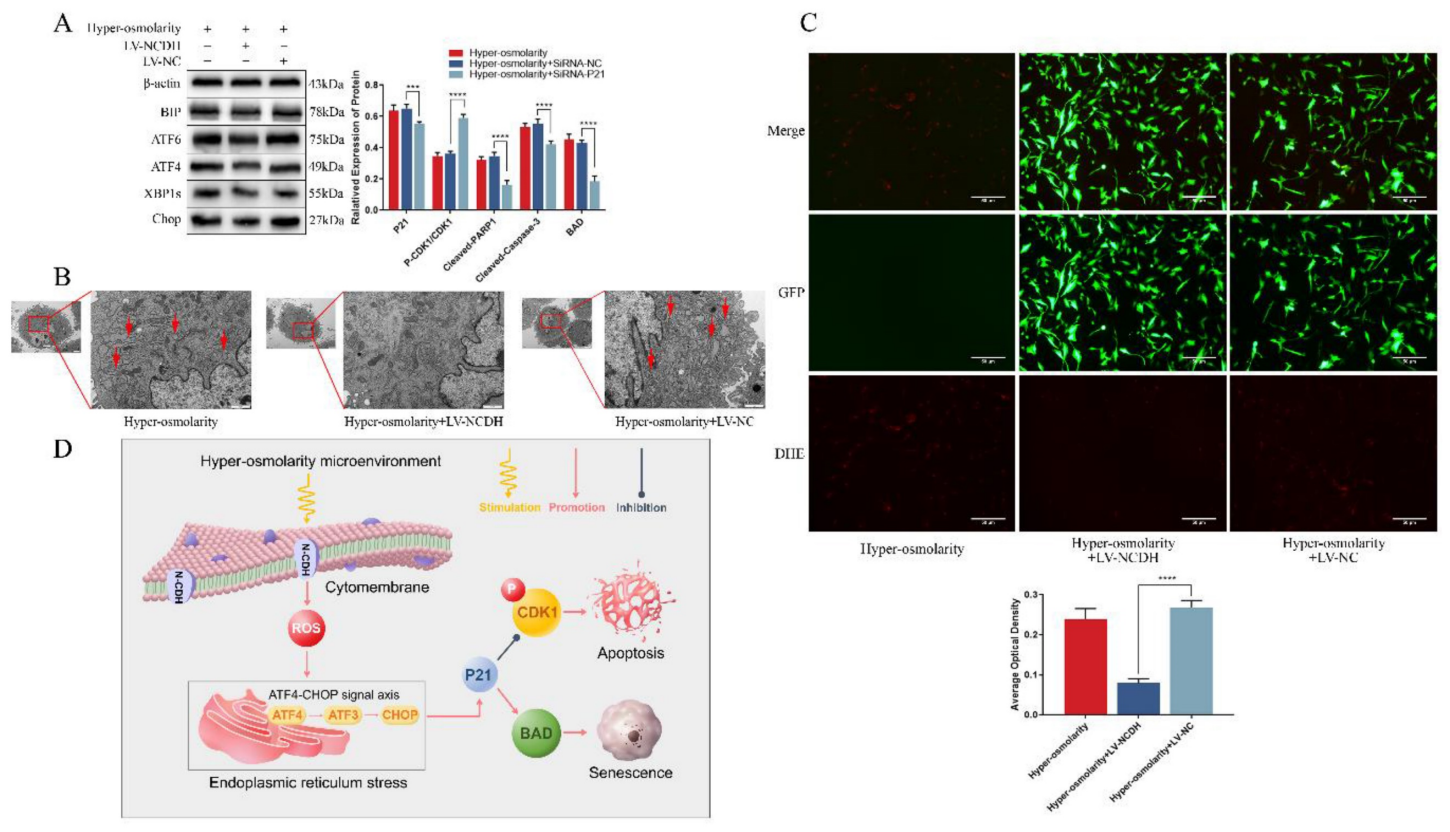
** ATF4-CHOP axis is a key signaling transduction pathway behind the protective effects of NCDH overexpression against endoplasmic reticulum stress of NPCs in the hyper-osmolarity microenvironment.** (A): Western blot analysis of protein expression of BIP, CHOP, ATF4, ATF6 and XPB1s in NPCs before and after NCDH overexpression in the hyper-osmolarity culture. (B): TEM analysis of ER morphology (red arrow) of NPCs after NCDH overexpression. Scale bars: 1 μm. (C): DHE probe staining analysis of ROS content in NPCs after NCDH overexpression. Scale bars: 50 μm. Note: The data represents mean ± SD (n=3); (D): Schematic diagram illustrates the protective effects of NCDH against a hyper-osmolarity microenvironment-induced undesirable cell fate of NPCs. ****p<0.0001 vs. hyper-osmolarity with LV-NC group. NPCs: nucleus pulposus cells, TEM: transmission electron microscopy, ER: endoplasmic reticulum, ROS: reactive oxygen species, DHE: dihydroethidium.

It was found in this study that apoptosis and senescence of NPCs were downstream events of ROS, ERS and P21 (Fig. 3A and Fig. 4A), and that ERS of NPCs was ROS-dependent (Fig. 3A-B) in the hyper-osmolarity microenvironment. To explore the effects of NCDH overexpression on ROS-dependent ERS, we used TEM and DHE probe to measure ROS and ERS of NPCs treated with LV-NCDH or LV-NC in a hyper-osmolarity microenvironment. According to the results, ERS and ROS levels were remarkably decreased in the LV-NCDH group (Fig. 6B-C).

Moreover, P21-regulated cell cycle arrest and cellular apoptosis was affected by ERS-related signal transduction 38. Hence, we further investigated the role of ATF4-CHOP axis (a key signaling transduction pathway involved in ERS) in the protective effects of NCDH overexpression in NPCs in the hyper-osmolarity microenvironment. The results of our study revealed that in relative to the LV-NC group, the expression of ERS markers (ATF4, ATF6, XPB1s, BIP and CHOP) decreased significantly in the LV-NCDH group (Fig. 6A). In light of that all these ERS markers were considered synergistic regulators of the ATF4-CHOP axis 44, we proposed that ATF4-CHOP axis was a key signaling transduction pathway behind the protective effects of NCDH overexpression in the hyper-osmolarity microenvironment (Fig 6D).

### NCDH overexpression alleviates ERS and inhibits NP degeneration of rat tail disc in vivo

**Figure 7:**
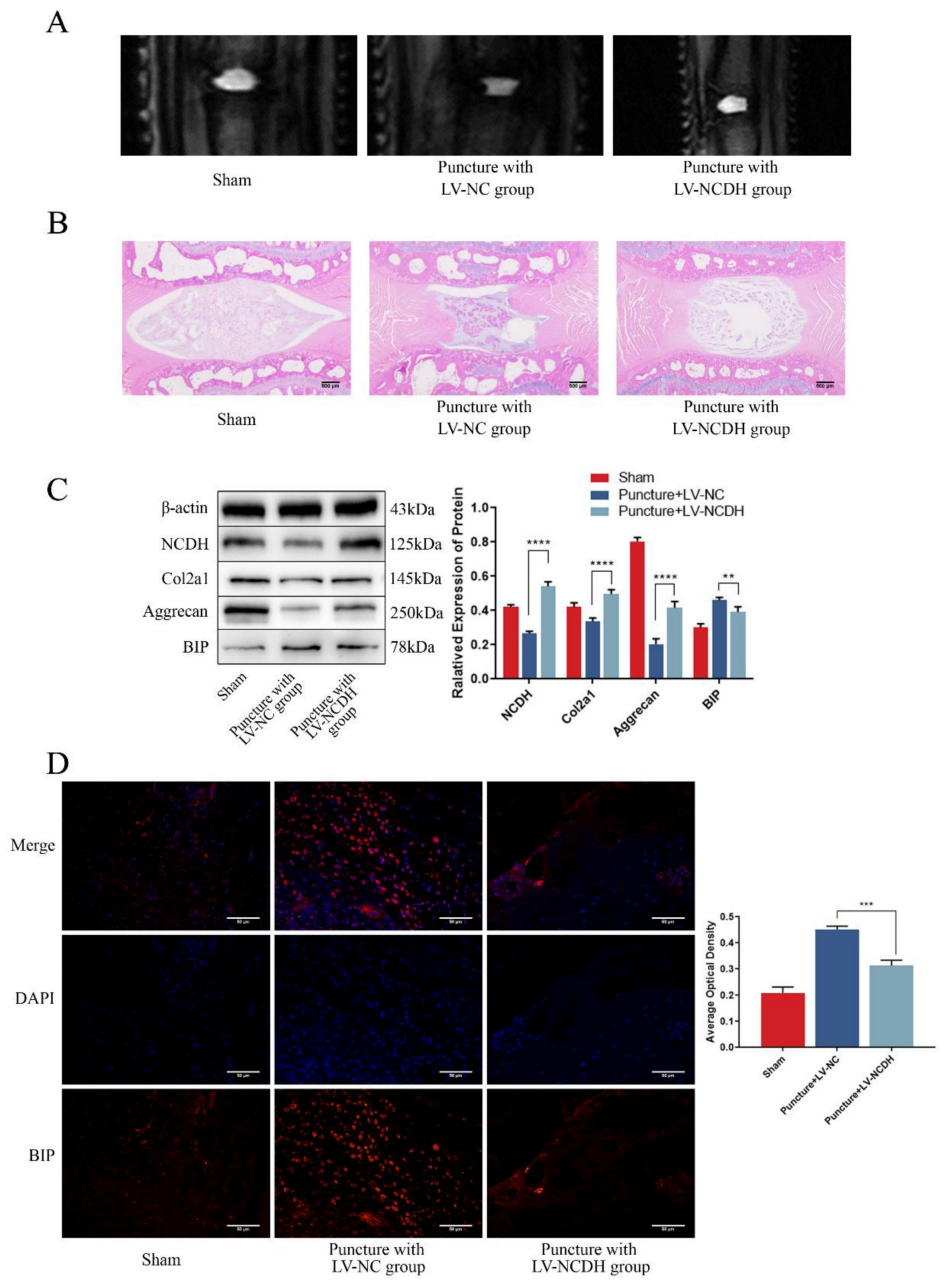
** NCDH overexpression alleviates ERS and inhibits NP degeneration of rat tail disc in vivo.** (A): T2-weighted MRI image of the experimental rat tail IVD. (B): HE staining of rat tail disc. Scale bars: 500 μm. (C): Western bolt analysis of protein expression of NCDH, Col2a1, Aggrecan and BIP. (D): Immunofluorescence staining of BIP expression. Scale bars: 50 μm. Note: The data represents mean ± SD (n=3); **p<0.01, ***p<0.001, ****p<0.0001 vs Puncture with LV-NC group.

To verify the protective effects of NCDH against ERS and NP degeneration in vivo, a rat tail puncture model was devised. The MRI scans indicated that the T2-weighted signal intensity of the LV-NCDH group (puncture with LV-NCDH group) exhibited a conspicuous elevation in comparison to the LV-NC group (puncture with LV-NC group) (Fig. 7A). After LV-NCDH treatment, damage to tissues and the extracellular matrix was less than that in animals treated with LV-NC (Fig. 7B). The western blot analysis substantiated the efficacy of LV-NCDH overexpression* in vivo* (Fig. 7C). Furthermore, the LV-NCDH group showed a lower expression level of BIP but a higher expression level of Aggrecan and Col2a1 than LV-NC group (Fig. 7C). In addition, immunofluorescence analysis showed that LV-NCDH treatment alleviated the increase in the expression of ERS marker (BIP) during IDD (Fig. 7D). Together, these results suggested that NCDH could alleviate ERS and NP degeneration *in vivo*.

## Discussion

Mechanical stress is an important factor that aggravates IDD 4, 45. However, the indirect effects and related mechanisms of mechanical stress need to be further investigated to develop specific therapeutic treatments. The preliminary theory is that mechanical stress promotes IDD by squeezing liquid from the intervertebral disc to increase osmotic pressure of the in-situ NP region 5. Therefore, investigations on the effects and the potential mechanisms of the hyper-osmolarity on NPCs are necessary to fully clarify the pathogenesis of IDD.

According to previous reports, the hyper-osmolarity microenvironment induces apoptosis and senescence through activating ROS in NPCs 6, 7. ROS are oxygen byproducts that have crucial roles in cell signaling and homeostasis, but excessive amounts can result in harm 46. Previously, it was believed that oxidative stress did not participate in IDD. In contrast, a series of recent studies have verified that intervertebral disc cells are capable of oxygen metabolism in vivo and generate ROS in the microenvironment 47, 48. Other evidence shows that ROS content is significantly increased in the degenerative intervertebral disc tissue 48. Importantly, ROS also induce ERS to synergistically damage cells 49, 50. Thus, ROS and ERS may be the key pathological processes during IDD. In the clinical disc samples, our results showed that ERS, apoptosis and senescence of NPCs were significantly aggravated, whereas the expression of cellular marker NCDH was obviously decreased. Therefore, we hypothesized that NCDH might affect disc cell activity through the ROS-ERS synergism in the hyper-osmolarity microenvironment.

ROS-ERS synergism can promote senescence or apoptosis with mitochondrial damage in human NPCs 8, 9. To investigate how the hyper-osmolarity microenvironment affects NPCs activity, we configured a hyper-osmolarity cell culture medium (550 mOsm/kg) to simulate the in-situ NP hyper-osmolarity microenvironment in vivo 6. In this study, senescence and apoptosis levels of NPCs were increased with the activation of ROS-ERS synergism, which was demonstrated by western blot, DHE fluorescent probes, transmission electron microscopy and SA-β-Gal staining in the hyper-osmolarity group. These findings suggested that the hyperosmolar microenvironment induced ROS-ERS synergism, apoptosis and senescence in rat NPCs. There was a complex interaction between oxidative stress and ERS. For example, Cayratidine increased ROS content though activating ERS which further forms a vicious cycle of ROS production in thymic cancer 13. Our results showed that ERS, apoptosis and senescence were attenuated after the inhibition of ROS with NAC. In contrast, apoptosis and senescence were decreased after the inhibition of ERS with 4-PBA, but the ROS level remained unchanged in the hyper-osmolarity microenvironment. Those above results revealed that the hyper-osmolarity microenvironment induced apoptosis and senescence of NPCs through ROS-dependent ERS. In our study, neither NAC nor 4-PBA could alleviate the downregulation of NCDH in the hyper-osmolarity microenvironment. Therefore, NCDH may be an upstream regulator of ROS-dependent ERS regarding apoptosis and senescence of NPCs in the hyper-osmolarity microenvironment.

Recent studies have indicated that NCDH is a typical cellular marker that maintains the cellular phenotype of NPCs and that its expression in NPCs is significantly decreased during IDD 51, 52. Our previous study showed that enhancing NCDH expression could effectively alleviate apoptosis and senescence of NPCs under overloaded compression 53. In the present study, we discovered that NCDH overexpression reversed apoptosis and senescence of NPCs by inhibiting ROS-dependent ERS in the hyper-osmolarity microenvironment, which was indicated by the decrease in ROS generation and expression of ERS-associated proteins (i.e. BIP, ATF4, ATF6, XBP1s and CHOP). ERS cause the accumulation of misfolded proteins when protein folding demand exceeds capacity in the endoplasmic reticulum 54.

When ERS occurred, IRE1 dissociated from glucose-regulated protein 78 (GRP78/BIP) to cleave X-box binding protein 1 (XBP1) mRNA and form active X-box binding protein 1 splice (XBP1s), which was further translated into the XBP1 protein 55. XBP1 induced the expression of rapamycin target protein (mTOR) and CCAAT/enhancer binding protein to promote the degradation of misfolded endoplasmic reticulum proteins and maintain intracellular protein homeostasis 56. Previously, these signaling pathways were considered to operate independently, but recent opinion asserts that the unfolded protein response (UPR) cooperates to complete ERS via the ATF4-CHOP axis 24, 44.

In this study, we presented a further discovery that the ATF4-CHOP axis was regulated by NCDH, which was indicated by decreased expression level of ATF4, ATF6, CHOP after NCDH overexpression. Recent studies have shown that the ATF4-CHOP axis induces senescence through G2/M phase arrest and apoptosis by activating the key target P21 in glioblastoma cells 38. Our results demonstrated that the relative expression of P21 were notably reduced while p-CDK1 (Thr-161) was significantly increased after NCDH overexpression which reversed the cell cycle arrest and cell viability suppression under hyper-osmolarity microenvironment. The CDK1 phosphorylation (Thr-161) was a key process for CDK1/Cyclin B complex formation 57, and inhibited G2/M phase arrest 38, 39. The drastic cell cycle change had irreversible consequences for the phenotypic maintenance of NPCs, which ultimately led to senescence 42, 58. Similarly, the cell viability suppression is also considered to be highly associated with the senescence of NPCs degeneration 59. In addition, our study indicated that the expression of Bad and Cleaved-PARP1 was lowered after LV-NCDH-mediated inhibition of P21. Furthermore, the P21-Bad signaling axis activated mitochondrial apoptosis by producing Cleaved-PARP1 and Cleaved-Caspase-3 39, 60. Combined with the flow cytometry and western blot results, we found that the hyper-osmolarity microenvironment activated the P21-Bad axis to induce apoptosis of NPCs. Together, P21 was a key target for ATF-4-CHOP signal axis to regulate the undesirable fates (apoptosis and senescence) of NPCs. These results suggested that NCDH inhibited ROS-dependent ERS-mediated apoptosis and senescence via suppressing the ATF4-CHOP axis in the hyper-osmolarity microenvironment. Similarly, the rat tail puncture injection model also showed that NCDH transfection reduced expression of the ERS-triggered protein BIP and inhibited the destruction of the extracellular matrix to mitigate IDD in vivo.

## Conclusions

To conclude, we investigated the impacts of NCDH on hyper-osmolarity microenvironment-induced senescence and apoptosis of NPCS, as well as the potential mechanism behind this process. Our results demonstrate that NCDH alleviates senescence and apoptosis of NPCs through inhibiting ROS-dependent ERS via the ATF4-CHOP axis in the hyper-osmolarity microenvironment. This study further clarifies pathogenesis of IDD and provides important mechanistic insights for biological treatment of IDD in the future clinical practice.

## Supplementary Material

Supplementary figures and tables.

## Abbreviations

NPCs: nucleus pulposus cells
NCDH: N-cadherin
ERS: endoplasmic reticulum stress
ROS: Reactive oxygen species
IDD: intervertebral disc degeneration
NP: nucleus pulposus
AGEs: advanced glycation end products
LVF: lumbar vertebral fracture
SD: Sprague-Dawley
LV: lentivirus vector
RNAi: RNA interference
siRNA: small interfering RNA
SA-β-Gal: senescence-associated β-Galactosidase
PBS: phosphate buffer saline
DHE: dihydroethidium
SDS: sodium dodecyl sulfate
PVDF: polyvinylidene difluoride
TEM: transmission electron microscopy
NC: negative control
NAC: N-acetylcysteine
4-PBA: sodium phenylbutyrate
MRI: magnetic resonance imaging
EMC: extracellular matrix macromolecules
G2/M: Gap 2/Mitosis
