# Supplementary material for: N-cadherin Alleviates Apoptosis and Senescence of Nucleus Pulposus Cells via Suppressing ROS-dependent ERS in the Hyper-osmolarity Microenvironment
Source: Int J Med Sci. 2024 Jan 1;21(2):341–56. doi: 10.7150/ijms.90591 (PMC10758147; doi:10.7150/ijms.90591)
Supplement: Supplementary file 1 — Supplementary figures and tables. [file ijmsv21p0341s1.pdf]

Supplementary material

Table S1. Basic information of patients

LVF	Age	Gender	Disc level of investigated	Degree of degeneration	Use
1	65	M	T12/L1	Pfirrmann I-II	Tissue and cell research
2	47	M	L1/2	Pfirrmann I-II	Tissue and cell research
3	76	M	L1/2	Pfirrmann I-II	Tissue and cell research
4	56	M	L4/5	Pfirrmann I-II	Tissue and cell research

IDD	Age	Gender	Disc level of investigated	Degree of degeneration	Use
1	46	F	L5/S1	Pfirrmann IV	Tissue research
2	31	M	L5/S1	Pfirrmann IV-V	Tissue and cell research
3	39	M	L4/5	Pfirrmann IV	Tissue research
4	65	M	L4/5	Pfirrmann IV	Tissue and cell research
5	40	F	L5/S1	Pfirrmann IV	Tissue and cell research
6	56	F	L5/S1	Pfirrmann IV	Cell research
7	71	M	L5/S1	Pfirrmann V	Tissue and cell research
8	46	F	L5/S1	Pfirrmann IV	Cell research
9	24	M	L5/S1	Pfirrmann IV	Tissue and cell research
10	48	F	L4/5, L5/S1	Pfirrmann IV	Tissue research
11	48	M	L4/5	Pfirrmann IV	Tissue research
12	74	F	L4/5, L5/S1	Pfirrmann IV-V	Tissue research
13	48	F	L5/S1	Pfirrmann IV	Tissue research
14	44	F	L5/S1	Pfirrmann V	Tissue and cell research
15	66	F	L3/4,L4/5	Pfirrmann IV-V	Tissue research
16	56	F	L4/5, L5/S1	Pfirrmann IV-V	Tissue and cell research
17	45	F	L5/S1	Pfirrmann IV-V	Tissue research
18	51	F	L4/5, L5/S1	Pfirrmann V	Cell research
19	48	M	L4/5	PfirrmannIV-V	Cell research
20	67	M	L4/5	PfirrmannV	Tissue research
21	44	M	L5/S1	Pfirrmann IV	Tissue research
22	20	F	L5/S1	PfirrmannV	Tissue and cell research
23	64	F	L5/S1	PfirrmannV	Tissue research
24	38	M	L4/5	PfirrmannIV-V	Tissue and cell research
25	55	M	L4/5	PfirrmannIV-V	Tissue and cell research

Figure S1. Effects of different osmotic pressures on rat nucleus pulposus cells (NPCs). The current hyper-osmolarity model of NPCs is achieved by controlling osmotic pressure through high glucose cultivation, but high glucose cultivation itself is one of the promoting factors for the degeneration of NPCs. To avoid the degenerative effects of high glucose cultivation on NPCs, we used NaCl to prepare an ion-based

hyper-osmolarity culture medium (under normal physiological conditions of intervertebral discs, there is Na^+, Cl^-, and negatively charged groups on the glycosaminoglycan chains) to construct the hyper-osmolarity microenvironment model for NPCs.

The Calcein AM/PI staining results indicate that both the 430 mOsm/kg culture medium and the control group (normal complete culture medium, 330 mOsm/kg) can support normal cultivation of NPCs without causing cell damage. The excessive cell damage caused by the 650 mOsm/kg condition is not tolerable for subsequent experiments, while the 550 mOsm/kg condition is considered moderate for modeling, and this condition was used in subsequent in vitro experiments to simulate the hyper-osmolarity microenvironment in rat NPCs (**$P<0.01$, ****$P<0.0001$).

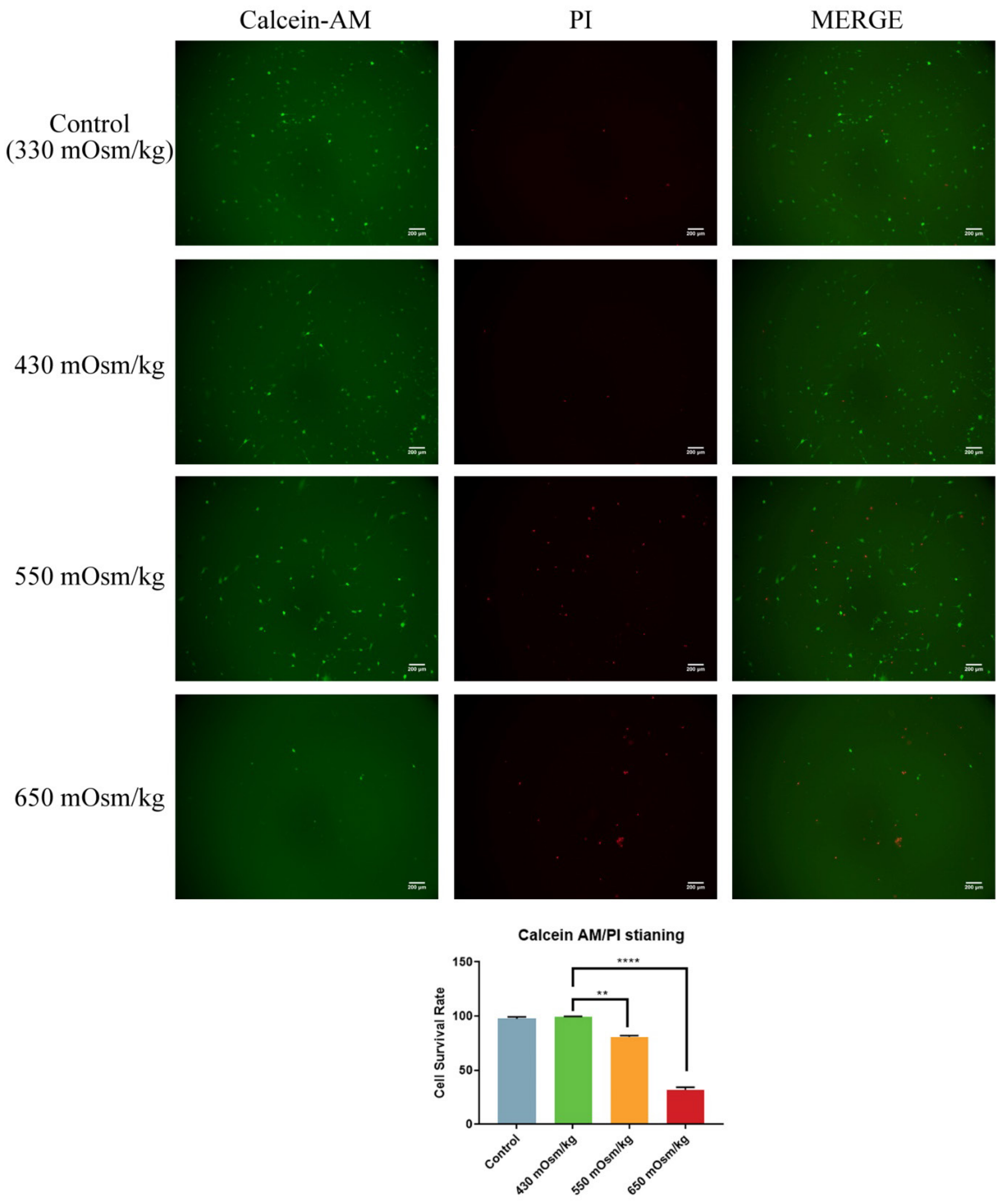
Calcein-AM
PI
MERGE
Control
(330 mOsm/kg)
430 mOsm/kg
550 mOsm/kg
650 mOsm/kg
Calcein AM/PI stianing
Cell Survival Rate
150
100
50
0
****
**
Control
430 mOsm/kg
550 mOsm/kg
650 mOsm/kg

Figure S2. Intervention of NCDH expression in rat NPCs under hyper-osmolarity microenvironment. Due to the low expression level of NCDH under hyper-osmolarity microenvironment, there was no statistically significant difference in NCDH expression between the RNAi-NCDH stable strain and the empty vector group under hyper-osmolarity microenvironment (NS, P>0.05). This suggests that RNAi-NCDH is unable to further interfere with NCDH expression under hyper-osmolarity microenvironment. Target sequence of RNAi-NCDH: 1. AAGCAGTGAACCAGCAGATTT; 2. GAGCCGATGAAGGAACCACAT; 3. ATCCCACTTACGGCCTTTCAA. Negative control insertion sequence: TTCTCCGAACGTGTCACGT.

Figure S3. Up-regulation of NCDH or intervention of P21 expression promoted the

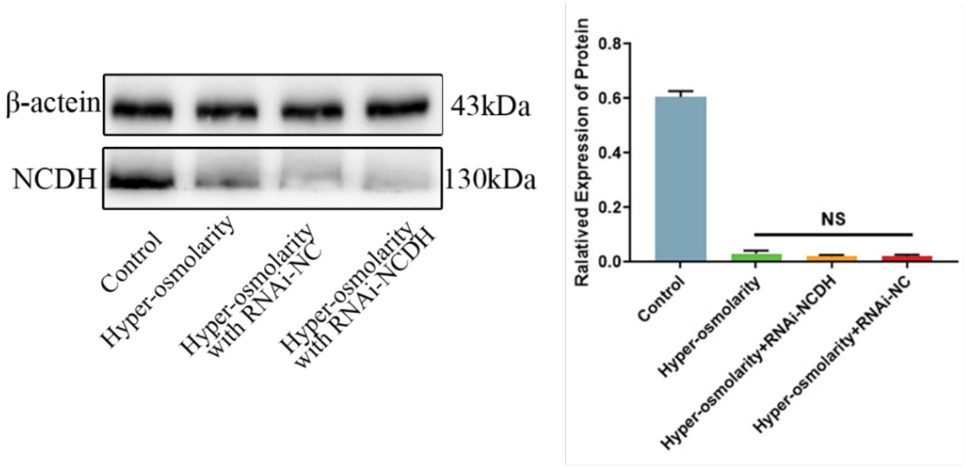

cell viability in rat NPCs under hyper-osmolarity microenvironment. (A): Compared to the control group, the cell viability of NPCs was significant decline in hyper-osmolarity microenvironment (***P<0.001 vs. control group). (B): Overexpression of NCDH inhibited the hyper-osmolarity microenvironment mediated cell activity decreasing in NPCs (**P<0.01 vs hyper-osmolarity with LV-NC group). (C): SiRNA-P21 enhanced the cell viability of NPCs under hyper-osmolarity microenvironment (**P<0.01 vs. hyper-osmolarity with SiRNA-NC group). Note: The data is collected from CCK-8 analysis and represents mean ± SD (n=3); NPCs: nucleus pulposus cells, CCK-8: Cell Counting Kit-8.

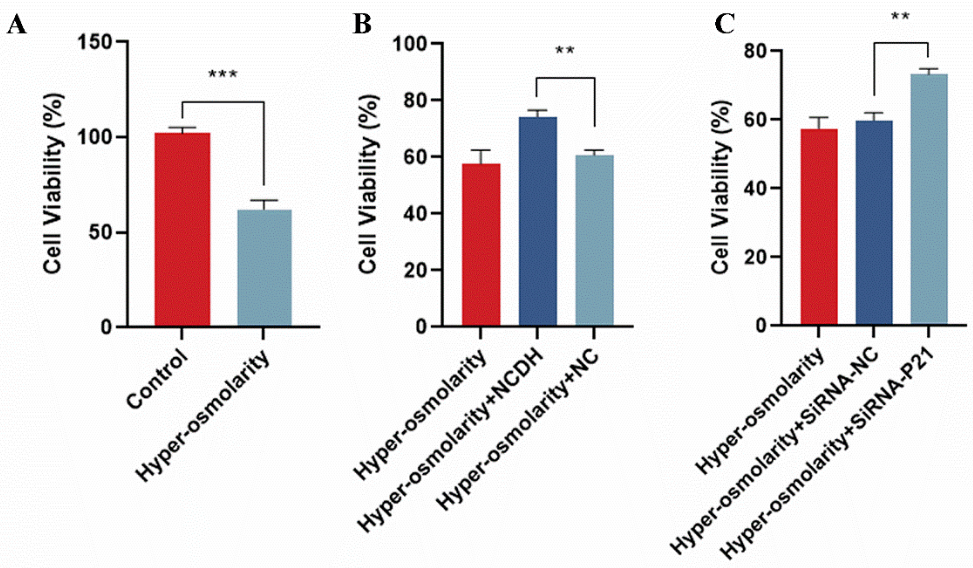
A
Cell Viability (%)
150
100
50
0
***
Control
Hyper-osmolarity
B
Cell Viability (%)
100
80
60
40
20
0
**
Hyper-osmolarity
Hyper-osmolarity+NCDH
Hyper-osmolarity+NC
C
Cell Viability (%)
80
60
40
20
0
**
Hyper-osmolarity
Hyper-osmolarity+SiRNA-NC
Hyper-osmolarity+SiRNA-P21
